# Integrated longitudinal analysis of ctDNA, radiologic response, and tumor volume reveals spatial and temporal heterogeneity in advanced melanoma

**DOI:** 10.1186/s12967-026-08899-0

**Published:** 2026-09-17

**Authors:** Isabel Heidrich, Anna Streckenbach, Charlotte Rautmann, Hanna Freiberg, Julian Kött, Glenn Geidel, Alessandra Rünger, Tim Zell, Carmen Roeper, Inga Hansen-Abeck, Finn Abeck, Stefan W. Schneider, Daniel J. Smit, Klaus Pantel, Christoffer Gebhardt

**Affiliations:** 1 Institute of Tumor Biology, Fleur Hiege Center for Skin Cancer Research, University Medical Center Hamburg-Eppendorf, Hamburg, Germany; 2 Department of Dermatology and Venereology, Fleur Hiege Center for Skin Cancer Research, University Medical Center Hamburg-Eppendorf, Hamburg, Germany; 3 Mildred Scheel Cancer Career Center HaTriCS4, University Medical Center Hamburg-Eppendorf, Hamburg, Germany; 4 Department of Diagnostic and Interventional Radiology and Nuclear Medicine, University Medical Center Hamburg-Eppendorf, Hamburg, Germany

**Keywords:** ctDNA, Longitudinal study, Melanoma, Liquid biopsy, Radiology, Volume-based analysis

## Abstract

**Background:**

In advanced melanoma, longitudinal disease monitoring remains limited by infrequent biomarker assessment and predominantly categorical imaging readouts. Although circulating tumor DNA (ctDNA) correlates with tumor burden, its behavior under high-frequency sampling and its relationship to quantitative metastatic tumor volume are poorly defined. Clarifying these dynamics is essential to establishing ctDNA as a clinically actionable tool for real-time treatment monitoring.

**Methods:**

We retrospectively analyzed 42 patients with unresectable stage III/IV melanoma treated with immune checkpoint inhibitors. Plasma ctDNA was quantified longitudinally across 241 time points using a UMI-based amplicon next-generation sequencing (NGS) assay detecting BRAF, EGFR, KRAS, NRAS, and PIK3CA. We paired ctDNA measurements with radiologic staging and correlated them with response categories (*n* = 99 time points) and volumetric tumor burden (*n* = 73 time points) using nonparametric statistics and receiver operating characteristic (ROC) analyses, including stratification by the ctDNA-imaging time interval and descriptive assessment of discordant patterns.

**Results:**

ctDNA levels were significantly associated with radiologic response according to RECIST 1.1, increasing across worsening response categories (Spearman ρ = 0.31, 95% CI 0.20–0.52, *p* = 0.002). Discriminative performance for identifying progression was moderate (AUC = 0.69). Threshold analyses demonstrated a sensitivity–specificity trade-off, with higher ctDNA levels providing greater specificity (e.g. ≥25 mutant molecules (MM)/mL: specificity ≥ 83%, sensitivity ≤ 30%), while lower thresholds showed limited sensitivity and specificity (e.g. 1–2 MM/mL: ~56% each). ctDNA concentrations were positively associated with total tumor volume (ρ = 0.46, *p* < 0.0001), with stronger correlations observed for temporally aligned measurements (0–30 days: ρ = 0.56). In contrast, dynamic changes in ctDNA were not significantly correlated with changes in tumor volume (ρ = 0.20, *p* = 0.25). Organ-associated analyses demonstrated marked heterogeneity in ctDNA shedding, with stronger associations in lymph node and peritoneal metastases and limited detectability in lung, liver, and brain metastases. Concordance between ctDNA dynamics and radiologic response was high in responding disease (CR/PR: 85.0%) but lower in stable or progressive disease (72.9%), with discordant cases associated with tumor heterogeneity, timing differences, and treatment-related factors.

**Conclusions:**

ctDNA reflects radiologic disease status and tumor burden, with increasing levels observed as response worsens. Despite heterogeneity in ctDNA shedding across metastatic sites, its significant correlation with tumor volume, particularly when closely timed to imaging, supports the potential value of ctDNA as a complementary biomarker for longitudinal disease monitoring while highlighting important biological and methodological factors that influence its clinical interpretation.

**Supplementary Information:**

The online version contains supplementary material available at https://doi.org/10.1186/s12967-026-08899-0.

## Introduction

Cutaneous melanoma remains one of the most aggressive solid tumors, despite major therapeutic advances through the introduction of immune checkpoint inhibitors (ICI) and targeted therapies [1]. A central clinical challenge is the early identification of patients at high risk of relapse or progression and the timely distinction between treatment response, pseudoprogression, and actual therapeutic failure [2]. Conventional markers such as LDH and S100, as well as imaging, often lack sufficient sensitivity to capture subtle changes in tumor burden [3, 4]. Consequently, circulating tumor DNA (ctDNA) has emerged as a promising minimally invasive biomarker for disease monitoring in melanoma.

Numerous studies have demonstrated that ctDNA correlates with tumor burden, provides independent prognostic information, and predicts progression-free and overall survival in metastatic melanoma [2, 5–9].

Longitudinal studies have further shown that early ctDNA kinetics during immune checkpoint inhibition and targeted therapy are associated with radiologic response and clinical outcome [10–13]. More recently, tumor-informed ctDNA assays have extended the clinical application of liquid biopsy toward molecular residual disease detection, further highlighting ctDNA’s potential for individualized melanoma management [2, 5, 12, 14, 15].

Despite this progress, longitudinal ctDNA assessment remains highly heterogeneous across studies. Sampling intervals often vary, with blood draws frequently aligned to clinical visits rather than standardized protocols. Importantly, very few studies use a consistent monthly sampling interval, even though this approach could provide clinically relevant granularity while remaining feasible in routine care. As a result, the evolution of ctDNA under strictly regular longitudinal monitoring across melanoma stages and its concordance with radiological staging remain insufficiently characterized. Discordant patterns, such as rising ctDNA despite stable imaging or detectable ctDNA in radiologically disease-free patients, may be clinically significant but have not been systematically explored.

To address this gap, the present study systematically evaluates longitudinal ctDNA measurements obtained at approximately four-week intervals in a melanoma cohort. It compares these trajectories with temporally matched staging assessments. By integrating serial ctDNA measurements with temporally matched RECIST assessments and three-dimensional volumetric tumor burden analyses, we investigated temporal concordance, organ-associated differences in ctDNA shedding, and clinically relevant molecular–radiologic discordance to define better the strengths and limitations of ctDNA for longitudinal disease monitoring.

## Methods

### Patient cohort

This retrospective study included 42 patients with advanced melanoma who received ICI (combination of cytotoxic T-lymphocyte-associated protein 4 (CTLA-4) inhibitor Ipilimumab, (YERVOY®, Bristol-Myers Squibb) 3 mg/kg body weight and programmed cell death protein 1 (PD-1) inhibitor Nivolumab (Opdivo®, Bristol-Myers Squibb) 1 mg/kg body weight every 3 weeks or Nivolumab (Opdivo®, Bristol-Myers Squibb) monotherapy 480 mg every 4 weeks; PD-1 inhibitor Pembrolizumab monotherapy 200 mg every 3 weeks or 400 mg every 6 weeks (KEYTRUDA®, Merck & Co, MSD)) at the University Skin Cancer Center Hamburg, Germany. The cohort was previously published (Heidrich *et al. J Exp Clin Cancer Res (2025))*. Blood sampling was prospectively integrated into routine clinical visits and therefore generally followed the respective immunotherapy treatment schedules. Consequently, sampling intervals reflected routine clinical practice rather than a strictly protocol-defined schedule. Minor deviations occurred due to individual clinical circumstances, including treatment delays, intercurrent illness, or scheduling constraints. Patients were enrolled between March 2018 and February 2022. All patients provided informed consent to participate in this study, which the Ethics Committee of the Hamburg Medical Association approved (PV5392). Inclusion criteria were: (i) a confirmed diagnosis of unresectable cutaneous or mucosal melanoma stage III or stage IV according to American Joint Committee on Cancer (AJCC) staging and classification (8th edition), (ii) at least 18 years of age, and (iii) at least 6 months of clinical follow-up. We collected blood samples from patients before treatment initiation and at regular intervals during follow-up. We analyzed ctDNA from all collected samples. Sampling was scheduled at approximately 4-week intervals; however, due to individual clinical circumstances or technical limitations, longer intervals between consecutive samples occasionally occurred.

### ctDNA analysis

Whole blood samples were centrifuged twice (1. Cycle: 300 ×g, 10 min, 23 °C, 2. Cycle: 1800 ×g, 10 min, 23 °C), and plasma was stored at − 80 °C until cell-free DNA (cfDNA) extraction. cfDNA was extracted using the QIA-amp Circulating Nucleic Acid Kit (Qiagen, Valencia, CA, USA, Cat No. 55114) according to the manufacturer’s instructions by vacuum processing. cfDNA was quantified using a Qubit™ 4 Fluorometer (Invitrogen™, Cat. No. Q33238, Carlsbad CA,2008, USA) with the Qubit™ dsDNA HS Assay Kit (Invitrogen™, Cat. No. 32854). cfDNA libraries were generated using the beta version of the Plasma-SeqSensei™ SOLID CANCER RUO kit (Sysmex Inostics GmbH). The libraries were then quantified on a Bioanalyzer (Agilent, Santa Clara, CA, USA) and sequenced on a NextSeq 550 instrument (Illumina, San Diego, CA, USA). The sequencing data were analyzed using proprietary software packages developed by Sysmex Inostics. ctDNA analysis was performed using the Plasma-SeqSensei™ Solid Cancer IVD kit (Sysmex Inostics), an amplicon-based assay specifically designed for high-sensitivity detection of somatic mutations in plasma-derived cfDNA. The input amount of cfDNA for library preparation ranged between 7.6 ng and 56.6 ng per time point per sample. We determined this individually for each time point based on cfDNA availability and quantification. The panel detects clinically relevant regions in *BRAF, EGFR, KRAS, NRAS,* and *PIK3CA* (Table 1).

**Table 1:** RefSeq IDs of BRAF, EGFR, KRAS, NRAS, and PIK3CA used to define the coding regions

Gene	Ensembl ID	RefSeq ID
*BRAF*	ENST00000288602.6	NM_004333.6
*EGFR*	ENST00000275493.2	NM_005228.5
*KRAS*	ENST00000256078.4	NM_033360.4
*NRAS*	ENST00000369535.4	NM_002524.5
*PIK3CA*	ENST00000263967.3	NM_006218.4

### Analytical sensitivity and specificity of ctDNA assay

The analytical performance of the assay was validated in accordance with CLSI guidelines (EP17–A2, EP05–A3, EP06-A, and EP07–A2). The limit of detection (LoD95) was determined as 6.21 mutant molecules (95% CI: 5.47–7.26), with a conservative reporting threshold set at ≥ 7 mutant molecules. We verified analytical specificity by in silico BLAST analysis to exclude cross-reactivity with the human genome and common microbial DNA. In addition, we established a position-specific limit of blank (LoB) using cfDNA from healthy donors and integrated it into the variant-calling algorithm to minimize false positives from background noise.

The analysis pipeline is based on SafeSEQ™ technology, which comprises three main steps: UID Family Grouping: Reads sharing the same Unique Molecular Identifier (UMI) are grouped to reduce random sequencing errors. Consensus Building: A consensus sequence is generated for each UID group, enhancing accuracy and reproducibility. Variant Calling: Consensus reads are aligned to the reference genome, and only variants above the defined LoD and outside the LoB are reported. Further technical specifications and validation data are available in the manufacturer’s Instructions for Use (IFU, ZR150537.R4) and via the Caresphere Academy’s Plasma-SeqSensei™ online training platform.

### Statistical analysis and data integration

Statistical analyses were performed in R (version ≥ 4.2) using RStudio, with data preprocessing conducted using dplyr, data. table, and readxl. The distribution of ctDNA values was assessed using the Shapiro–Wilk test. ctDNA values showed a non-normal, right-skewed distribution with the presence of outliers; therefore, non-parametric statistical methods were applied. Correlation analyses, including Spearman’s rank correlation, were performed using base R functions, while diagnostic performance was assessed using receiver operating characteristic (ROC) and precision-recall analyses implemented in the pROC and PRROC packages. Data visualization was performed using ggplot2, pheatmap, and finalfit. Pearson and Spearman correlations, as well as selected figures, were additionally generated using GraphPad Prism (version 10).

Radiologic responses were harmonized into four categories (CR, PR, SD, PD) and encoded on an ordinal disease-burden scale. We calculated aggregate ctDNA concentration at each time point as the sum of the mutation-specific values. For RECIST-based analyses, ctDNA measurements were matched to radiologic assessments within a ± 40-day window, resulting in paired ctDNA-staging observations. We selected a ± 40-day interval to balance temporal proximity with the number of evaluable ctDNA-imaging pairs in this retrospective real-world cohort, where imaging and blood sampling were not always performed on the same day due to routine clinical scheduling. Group-wise differences in ctDNA levels across response categories were assessed using the Kruskal–Wallis test with nonparametric post-hoc comparisons, and associations with ordinal response were quantified using Spearman’s rank correlation. For diagnostic analyses, responses were dichotomized into CR/PR versus SD/PD, and ctDNA was treated as a continuous predictor in ROC analyses, with confidence intervals estimated using bootstrap resampling with 1,000 iterations.

### Assessment of volumetric tumor burden

In contrast, volumetric tumor burden analyses were designed to maximize the number of evaluable ctDNA-imaging pairs because retrospective three-dimensional segmentation was only available for a subset of imaging examinations. Therefore, all available paired ctDNA-imaging observations were included using the nearest available CT examination, irrespective of the temporal interval between blood sampling and imaging. Because this approach resulted in variable ctDNA-imaging intervals (0–197 days), predefined sensitivity analyses were performed by stratifying paired observations according to the temporal distance between ctDNA sampling and imaging (0–30, 31–90, and >90 days). These analyses were intended to evaluate the influence of temporal alignment on the observed ctDNA–tumor volume correlations (Suppl. Table 4).

We assessed total volumetric tumor burden using three-dimensional segmentation of all measurable melanoma lesions on contrast-enhanced CT scans. Lesions were contoured on axial slices using a semi-automated workflow implemented in Sectra IDS7 and Syngo (Siemens Healthineers). Total tumor burden was defined as the cumulative volume of all segmented melanoma lesions across all anatomical sites; non-melanoma findings were excluded from the analyses. Lesions with a volume below 0.5 mL were considered below the predefined threshold for reliable and reproducible volumetric assessment and were therefore excluded from quantitative segmentation. Overall, we excluded eleven lesions on this basis, including four intracranial lesions, one pulmonary lesion, and six lymph node metastases. To ensure methodological consistency and minimize interobserver variability, two board-certified radiologists independently reviewed all segmentations using a four-eyes principle, with discrepancies resolved by consensus. In contrast to RECIST 1.1, which relies on one-dimensional measurements of selected target lesions, this approach provides a more comprehensive assessment of total tumor burden. Longitudinal changes in tumor volume were calculated between consecutive imaging time points and expressed as relative percentage change (Suppl. Figure 1).

## Results

### Patient cohort and distribution of ctDNA mutations

We analyzed plasma-derived ctDNA for the five different cancer-associated genes (*BRAF, EGFR, KRAS, NRAS, PIK3CA)* from 241 time points of 42 melanoma patients, of which 90.5% were in metastatic American Joint Committee on Cancer (AJCC) stage IV and 9.5% in unresectable AJCC stage III; 66.7% of these patients were male, and 33.3% were female [12]. The enrolled patients had a median age of 71 years (range, 39–87 years). In addition to cutaneous melanoma (*n* = 38), patients presented with mucosal melanoma (*n* = 4). 66.7% of patients received anti-CTLA-4/anti-PD-1 combination therapy (Ipilimumab + Nivolumab), 26.2% received Pembrolizumab monotherapy, and 7.1% received Nivolumab monotherapy. For most of the patients (76.2%) included in this study, first-line treatment was administered.

Overall, 40.5% of patients with available primary tumor tissue had a *BRAF* mutation in their tumor tissue (35.1% *BRAF*^V600E^, 2.7% *BRAF*^V600R^, *BRAF*^V600K^ 2.7%), 18.9% of patients had a *NRAS* mutation (10.8% *NRAS*^Q61R^, 8.1% *NRAS*^Q61K^) and one patient (2.8%) had a *KRAS* mutation *(KRAS*^*G12*^) in their tissue. No *PIK3CA* mutations were found in the tissue (Table 2). Blood samples were collected from 34 patients at baseline (start of treatment) and every 3–4 weeks thereafter, and from 8 patients at 12 weeks before and after progression during immunotherapy.

**Table 2:** Demographic, clinical, and pathological parameters of the study population

	Total N		N (%)
Sex	42 (100.0)	Male	28 (66.7)
		Female	14 (33.3)
Age group	42 (100.0)	<65	12 (28.6)
		≥65	30 (71.4)
Primary melanoma site	42 (100.0)	Cutaneous	38 (90.5)
		Mucosal	4 (9.5)
*BRAF* status (tissue)	37* (94.9)	Wildtype	22 (59.5)
		BRAF V600E	13 (35.1)
		BRAF V600K	1 (2.7)
		BRAF V600R	1 (2.7)
*KRAS* status (tissue)	36* (87.2)	Wildtype	33 (91.7)
		Mutated	1 (2.8)
*NRAS* status (tissue)	37* (94.9)	Wildtype	30 (81.1)
		NRAS Q61R	4 (10.8)
		NRAS Q61K	3 (8.1)
*PIK3CA* status (tissue)	36* (92.3)	Wildtype	36 (100.0)
AJCC	42 (100.0)	Stage III	4 (9.5)
		Stage IV	38 (90.5)
T	42 (100.0)	T0	10 (23.8)
		T1	3 (7.1)
		T2	6 (14.3)
		T3	6 (14.3)
		T4	15 (35.7)
		Tx	2 (4.8)
N	42 (100.0)	N0	16 (38.1)
		N1	9 (21.4)
		N2	9 (21.4)
		N3	8 (19.0)
M	42 (100.0)	M0	5 (11.9)
		M1	37 (88.1)
Baseline therapy	42 (100.0)	Ipilimumab + Nivolumab	28 (66.7)
		Pembrolizumab	11 (26.2)
		Nivolumab	3 (7.1)
No. of treatment lines	42 (100.0)	First line	32 (76.2)
		Second line	8 (19.0)
		Third line	2 (4.8)

*The patients missing here did not have enough tissue for mutational analysis

**EGFR analysis in tissue was not performed

T, tumor; N, lymph nodes; M, distant metastasis; according to the 8th edition of the AJCC cancer staging manual

### Associations of ctDNA with established melanoma biomarker

Exploratory analyses of established serum biomarkers demonstrated significantly higher baseline S100 concentrations in patients with detectable baseline ctDNA, whereas baseline LDH showed no significant association with ctDNA status. Neither S100 nor LDH was associated with progression-free or overall survival in subsequent univariable or multivariable analyses (Suppl. Tables 1-3).

### Longitudinal ctDNA dynamics

**Fig. 1:**
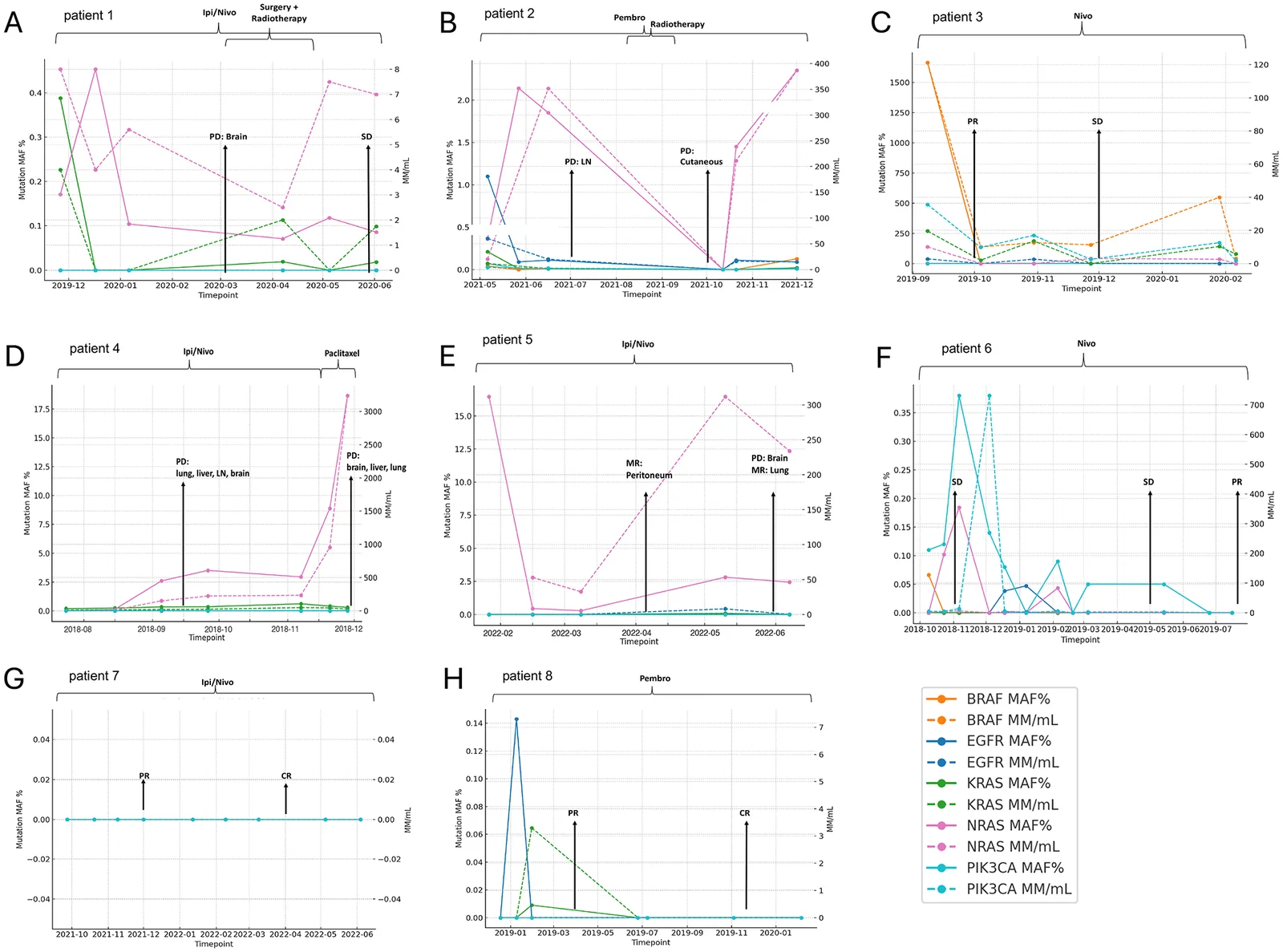
Representative longitudinal mutation-specific ctDNA trajectories under immune checkpoint inhibitor therapy. (**A**–**H**) Longitudinal ctDNA profiles of *n* = 8 patients illustrating distinct molecular–radiologic response patterns during treatment. For each panel, mutation-specific ctDNA allele frequencies (MAF,%, left ordinate) and absolute ctDNA concentrations (MM/mL, right ordinate) for *BRAF, EGFR, KRAS, NRAS,* and *PIK3CA* are shown over time. Radiologic staging events (PR, CR, SD, PD) are indicated by vertical arrows. Treatment regimens are annotated above each panel. Individual panels exemplify heterogeneous ctDNA kinetics, including molecular progression preceding radiologic PD/SD, molecular–radiologic discordance (elevated ctDNA during PR/CR or undetectable ctDNA during SD/PD), and sustained molecular remission during radiologic response (CR, PR). MR, mixed response: a mixed response is a heterogeneous treatment response, with some lesions regressing while others progress. CR, complete remission; MM*,* mutant molecules; PD, progressive disease; PR, partial remission; SD, stable disease

Longitudinal ctDNA profiling demonstrated pronounced inter-individual and mutation-specific heterogeneity. Representative patient trajectories illustrate diverse kinetic patterns including persistent low-level ctDNA, transient peaks preceding radiologic progression, and asynchronous fluctuations of multiple concurrent mutations (Fig. 1 A-H). Overall, patients contributed a median of 6 plasma samples (IQR 5–7.75), with a median sampling interval of 28 days (IQR 21–42), reflecting routine longitudinal monitoring during systemic treatment. Across additional representative cases, we observed distinct ctDNA response patterns. ctDNA levels increased early and were associated with subsequent disease progression, suggesting that ctDNA dynamics preceded radiologic progression in these cases (patients 1, 2, 4) (Figs. 1A, B, D). In contrast, two patients [3, 6] initially demonstrated a PR accompanied by declining ctDNA levels, followed by SD with persistently detectable ctDNA, consistent with ongoing tumor burden despite radiologic stabilization (Figs. 1C, F). Patients 7 and 8 showed rapid declines or absence of detectable ctDNA and exhibited early clinical response within the first three months after initiation of immunotherapy (Figs. 1G, H). Using a longitudinal, event-based analysis of serial ctDNA measurements, a decline in *BRAF* ctDNA was followed by a concomitant increase in *NRAS, KRAS*, or *EGFR* in 17 of 39 evaluable events (43.6%) (Figs. 1A, B, D, E). Patient 5 (Fig. 1E) exemplifies a mixed response (MR) pattern, characterized by regression of some lesions while new progressive disease emerged in the peritoneum. This spatially heterogeneous response was paralleled by increased ctDNA dynamics before radiologic detection of MR and progression. Treatment was continued due to the overall mixed response pattern. Full longitudinal profiles for all patients are provided in the Supplementary Material (Suppl. Figure 2).

### Association with radiologic response (RECIST 1.1)

**Fig. 2:**
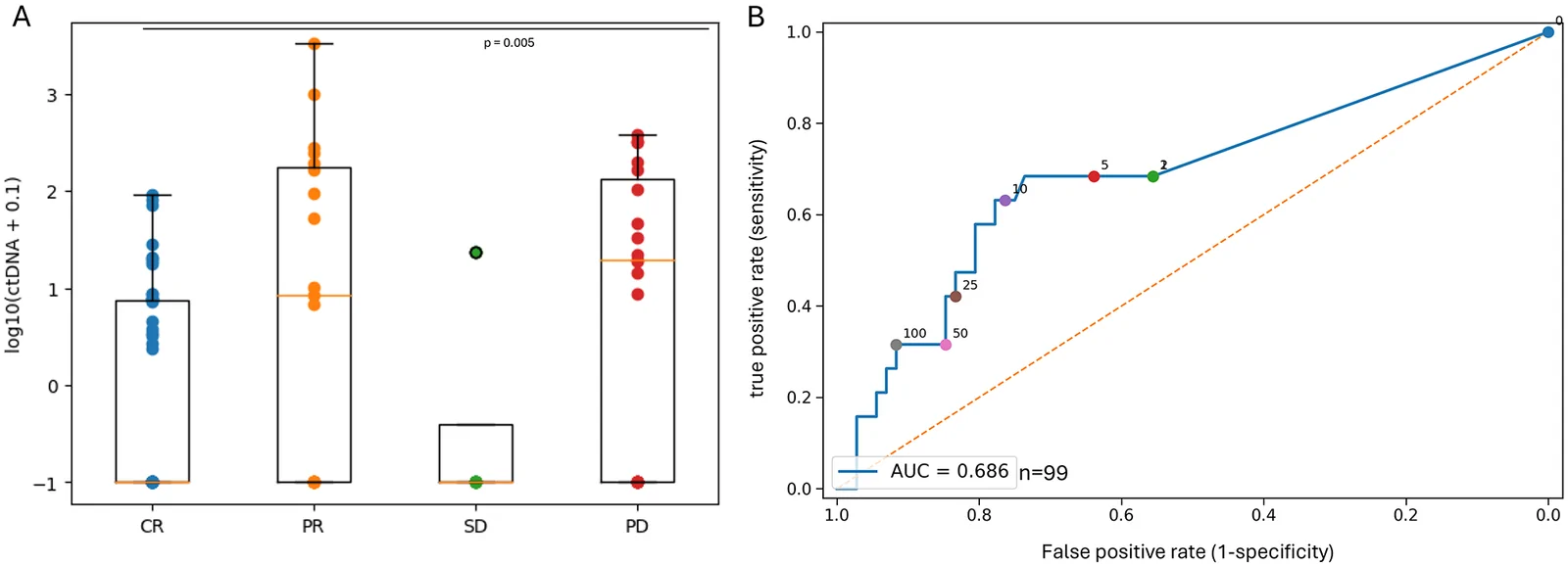
ctDNA levels across response categories and diagnostic performance for predicting progressive disease. (**A**) Distribution of ctDNA concentrations (MM/mL) across RECIST response categories (CR, PR, SD, PD) using ctDNA–radiology pairs matched within ±40 days (*n* = 99 time points, *n* = 27 PD/SD, *n* = 72 CR/PR cases, *n* = 34 patients). Each point represents one matched ctDNA–radiology pair. Boxplots display the median, interquartile range (IQR), and whiskers extending to 1.5 × IQR; outliers are shown individually. Group comparisons were performed using the Kruskal–Wallis test followed by nonparametric post-hoc pairwise comparisons with adjustment for multiple testing. (**B**) Receiver operating characteristic (ROC) curve assessing the ability of continuous ctDNA concentrations to discriminate progressive disease (PD/SD) from non-progressive disease (CR/PR). The area under the curve (AUC) and sample size (*n* = 99 matched ctDNA-radiology pairs) are indicated. Selected ctDNA thresholds are annotated to illustrate the corresponding sensitivity–specificity trade-offs. CR, complete remission; MM, mutant molecules; PD, progressive disease; PR, partial remission; SD, stable disease

Across all ctDNA–staging pairs matched within ±40 days (*n* = 99 time points, *n* = 27 PD/SD, *n* = 72 CR/PR cases, *n* = 34 patients), ctDNA levels differed significantly across radiologic response categories according to RECIST 1.1 (CR, PR, SD, PD; Kruskal–Wallis *p* < 0.001). ctDNA concentrations were lowest in CR and SD (median 0 Mutant molecules (MM)/mL), intermediate in PR (median 8.3 MM/mL), and highest in PD (median 19.3 MM/mL), with wide interquartile ranges (CR: 0–7.3; PR: 0 -177.7; SD: 0–5.9; PD: 0–135.0 MM/mL) (Fig. 2A). Post-hoc analyses demonstrated significantly elevated ctDNA levels in PD compared with CR (adjusted *p* = 0.005). No other pairwise comparisons remained statistically significant after correction, although a trend towards higher ctDNA levels in PR compared with CR was observed (adjusted *p* = 0.098) (Fig. 2A).

Spearman correlation analysis confirmed a significant monotonic association between increasing ctDNA levels and worsening radiologic response across the ordinal response scale (ρ = 0.31, 95% CI 0.20–0.52, *p* = 0.002) (Suppl. Table 4).

Consistent with the ROC analysis (Fig. 2B), predefined ctDNA thresholds demonstrated a clear trade-off between sensitivity and specificity. Low thresholds (1–2 MM/mL) provided balanced performance (sensitivity 56%, specificity 56%), corresponding to the mid-section of the ROC curve. Increasing thresholds shifted operating points along the curve toward higher specificity: at 10 MM/mL, specificity increased to 76% with a modest reduction in sensitivity (52%), while higher thresholds (≥25 MM/mL) achieved high specificity (≥83%) at the expense of markedly reduced sensitivity (≤30%). At the highest threshold (100 MM/mL), specificity reached 92% with minimal false positives, but sensitivity remained low (22%), reflecting substantial loss of true-positive detections (Table 3).

**Table 3:** Diagnostic performance of predefined ctDNA thresholds for identifying progressive or stable disease (PD/SD)

Threshold (MM/mL)	Sensitivity	Specificity	TP	FP	TN	FN
0	1.00	0.00	27	72	0	0
1	0.56	0.56	15	32	40	12
5	0.56	0.64	15	26	46	12
10	0.52	0.76	14	17	55	13
25	0.30	0.83	8	12	60	19
50	0.22	0.85	6	11	61	21
100	0.22	0.92	6	6	66	21

MM, mutant molecules; TP, positives; FP, false positives; TN, true negatives; FN, false negatives; Sensitivity, proportion of PD/SD cases correctly identified; Specificity, proportion of CR/PR cases correctly identified

### Volume-resolved analysis of ctDNA and tumor burden

**Fig. 3:**
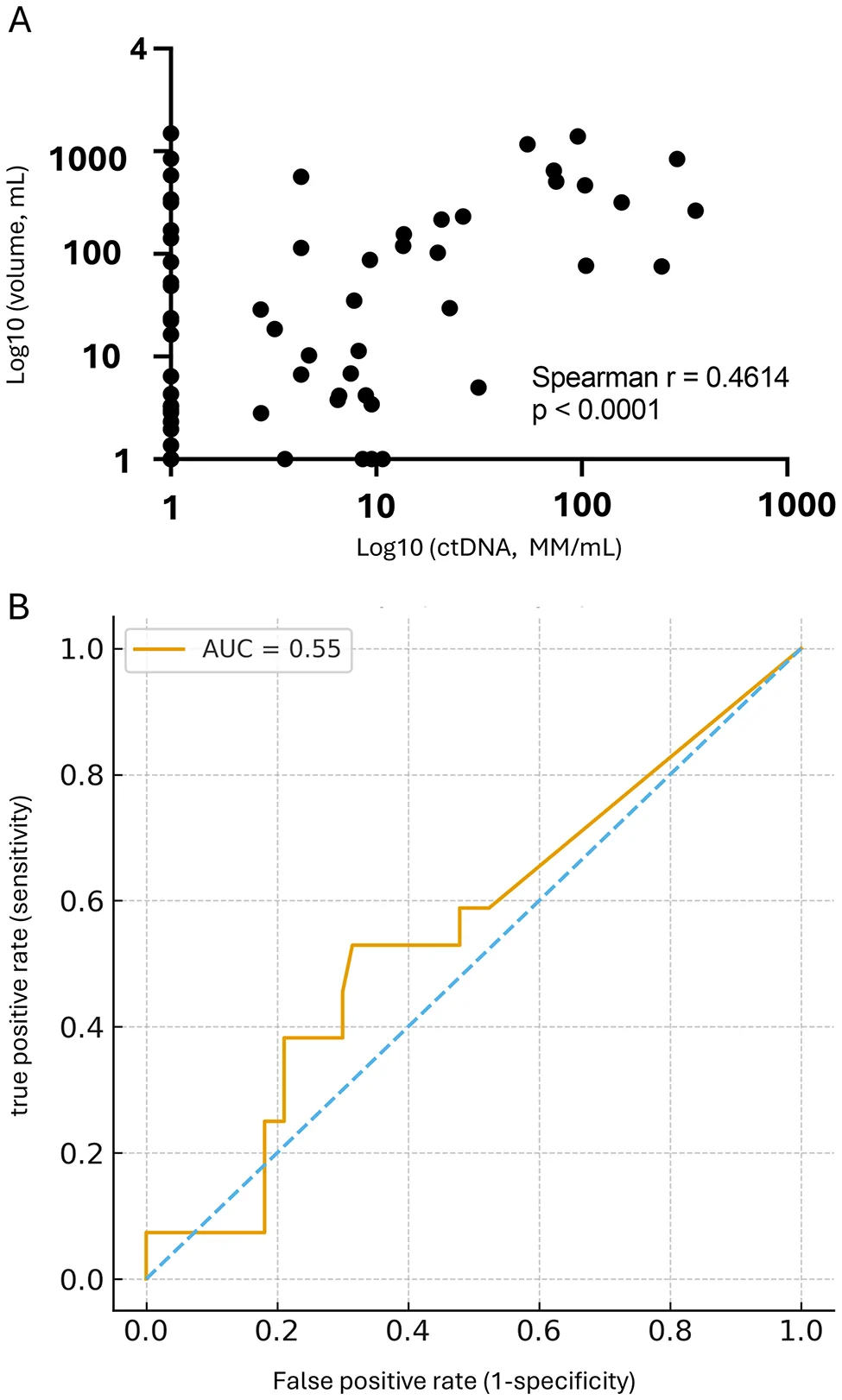
Association between ctDNA concentration and radiologically determined total metastatic tumor volume. (**A**) Scatter plot showing the association between log₁₀-transformed ctDNA concentration (MM/mL) and total tumor volume (mL) across all paired measurements (*n* = 73 paired measurements). A moderate positive correlation was observed between ctDNA concentration and total tumor volume (Spearman ρ = 0.46, *p* < 0.0001) (**B**) The AUC and sample size (*n* = 73 matched ctDNA-imaging pairs) are reported. ROC analyses were performed using logistic regression (AUC = 0.55)

To evaluate whether ctDNA reflects total metastatic tumor volume, we analyzed 73 paired ctDNA measurements and volumetric imaging assessments from 37 patients, spanning intervals of 0–197 days between blood sampling and radiologic imaging. ctDNA concentrations correlated significantly with total tumor volume (Spearman ρ = 0.46, 95% CI 0.25–0.63, *p* < 0.0001; Fig. 3A).

To assess whether temporal alignment between sampling and imaging influenced the observed associations, we performed sensitivity analyses across different intervals between ctDNA sampling and imaging. Sensitivity analyses demonstrated that this association depended on temporal alignment between blood sampling and imaging. Correlations were strongest for pairs obtained within 30 days (ρ = 0.56, *p* < 0.0001), weaker and non-significant for intervals of 30–90 days (ρ = 0.23, *p* = 0.29), and should be interpreted cautiously for intervals > 90 days because only eight paired measurements were available (Suppl. Table 5).

To evaluate whether dynamic changes in ctDNA levels reflect corresponding changes in total metastatic tumor volume, we assessed the association between changes in tumor volume (Δtumor volume) and changes in ctDNA concentration (ΔctDNA), and examined the influence of the time interval on these relationships. In contrast, longitudinal changes in ctDNA (ΔctDNA) were not significantly associated with changes in tumor volume (Δtumor volume) (ρ = 0.20, 95% CI −0.16 - 0.52, *p* = 0.25; Suppl. Table 3). While longer sampling intervals showed a trend toward greater discordance in Δtumor volume (ρ = −0.34, 95% CI −0.61 to −0.01, *p* = 0.051), ΔctDNA was not associated with sampling interval (Suppl. Table 6).

Finally, we evaluated the ability of ctDNA to discriminate volumetric disease states. ROC analysis demonstrated only moderate discrimination between increasing (PD) and decreasing or absent total metastatic tumor volume (PR/CR), with an area under the curve (AUC) of 0.55 (Fig. 3B). Bootstrap resampling revealed no significant difference compared with the RECIST-based analysis, consistent with the weak association between ΔctDNA and volumetric tumor changes. This is consistent with the observed lack of correlation between ΔctDNA and Δtumor volume.

### Site-specific associations between tumor volume and circulating ctDNA

**Fig. 4:**
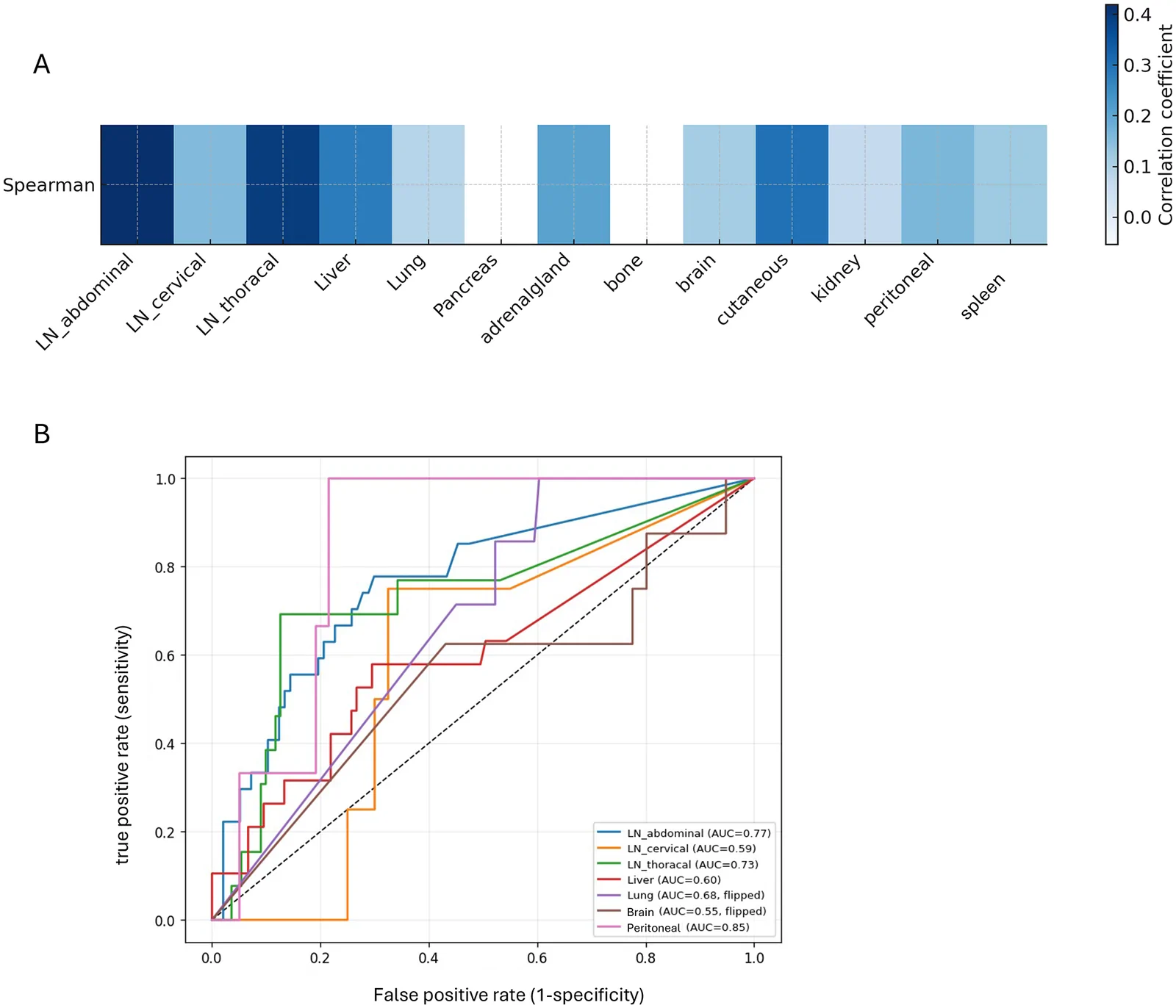
Association between ctDNA concentration and organ-associated volumetric tumor burden. (**A**) Heatmap showing Spearman’s rank correlation coefficients (ρ) between ctDNA concentration and organ-associated metastatic tumor volumes across anatomical sites. Color intensity reflects the strength of correlation. (**B**) Organ-associated differences in ctDNA shedding ROC curves showing the ability of ctDNA to discriminate volumetric disease states across metastatic sites. For most sites, higher ctDNA values defined positive cases (≥threshold), whereas for sites with inverse associations, classification was inverted (“flipped”; ≤threshold). The dashed line denotes chance-level performance (AUC = 0.5). N per anatomical site (applies to A and B): brain (*n* = 16), cervical lymph nodes (LN) (*n* = 8), thoracic lymph nodes (LN) (*n* = 29), abdominal lymph nodes (*n* = 32), liver (*n* = 32), peritoneal (*n* = 7), lung (*n* = 24), kidney (*n* = 2), adrenal gland (*n* = 28), cutaneous (*n* = 19), spleen (*n* = 6), bone (*n* = 0), and pancreas (*n* = 1). N refers to the number of organ-associated time point observations (i.e., instances where a given metastatic site was present and could be correlated with a concurrent ctDNA measurement), not the number of individual patients

To further dissect the relationship between ctDNA and total metastatic tumor volume, we next performed organ-associated analyses to assess how metastatic lesions in different anatomical sites contribute to circulating ctDNA levels. Most patients presented with metastases involving two to four anatomical sites (*n* = 33), whereas only five had a single metastatic site. Spearman correlations were strongest for thoracic (ρ = 0.42, 95% CI 0.08–0.61, *n* = 29) and abdominal lymph nodes (ρ = 0.39, 95% CI 0.06–0.52, *n* = 32), followed by cutaneous (ρ = 0.30, 95% CI −0.20–0.53, *n* = 19) and liver metastases (ρ = 0.28, 95% CI −0.09–0.66, *n* = 32), whereas lung (*n* = 24) and brain metastases (*n* = 16) showed weak or negligible correlations; bone metastases could not be assessed due to the absence of positive time points (*n* = 0) (Fig. 4A).

To further characterize this subgroup, a more detailed analysis of patients with brain metastases was performed. Patients with >3 metastases had significantly higher tumor volumes than those with 1–3 metastases (Mann–Whitney U test, *p* = 0.017). In contrast, ctDNA levels did not differ significantly between the two groups (*p* = 1.00), despite higher mean values observed in patients with fewer metastases. Tumor volume ranged from 0.33 mL to 1099.65 mL (Suppl. Figure 3).

ROC analyses likewise demonstrated marked heterogeneity across metastatic sites (Fig. 4B). The highest discriminative performance was observed for peritoneal (AUC = 0.85, *n* = 7), abdominal lymph node (AUC = 0.77, *n* = 32), and thoracic lymph node metastases (AUC = 0.73, *n* = 29), whereas pulmonary (AUC = 0.68, *n* = 24), liver (AUC = 0.60, *n* = 32), and cervical lymph node metastases (AUC = 0.59, *n* = 8) showed only moderate performance. Brain metastases demonstrated the lowest discriminative accuracy (AUC = 0.55, *n* = 16).

Because brain metastases showed the weakest association with ctDNA, we performed an exploratory subgroup analysis (Suppl. Figure 3). One patient presented with a single metastasis at baseline that was too small to measure, and another patient developed a single metastasis during treatment (1.85 mL). In both cases, ctDNA was not detectable (time interval: 6–11 days). Three patients had brain metastases with one to three additional metastatic sites at baseline, while two patients had brain metastases with three or more additional sites at baseline.

**Fig. 5:**
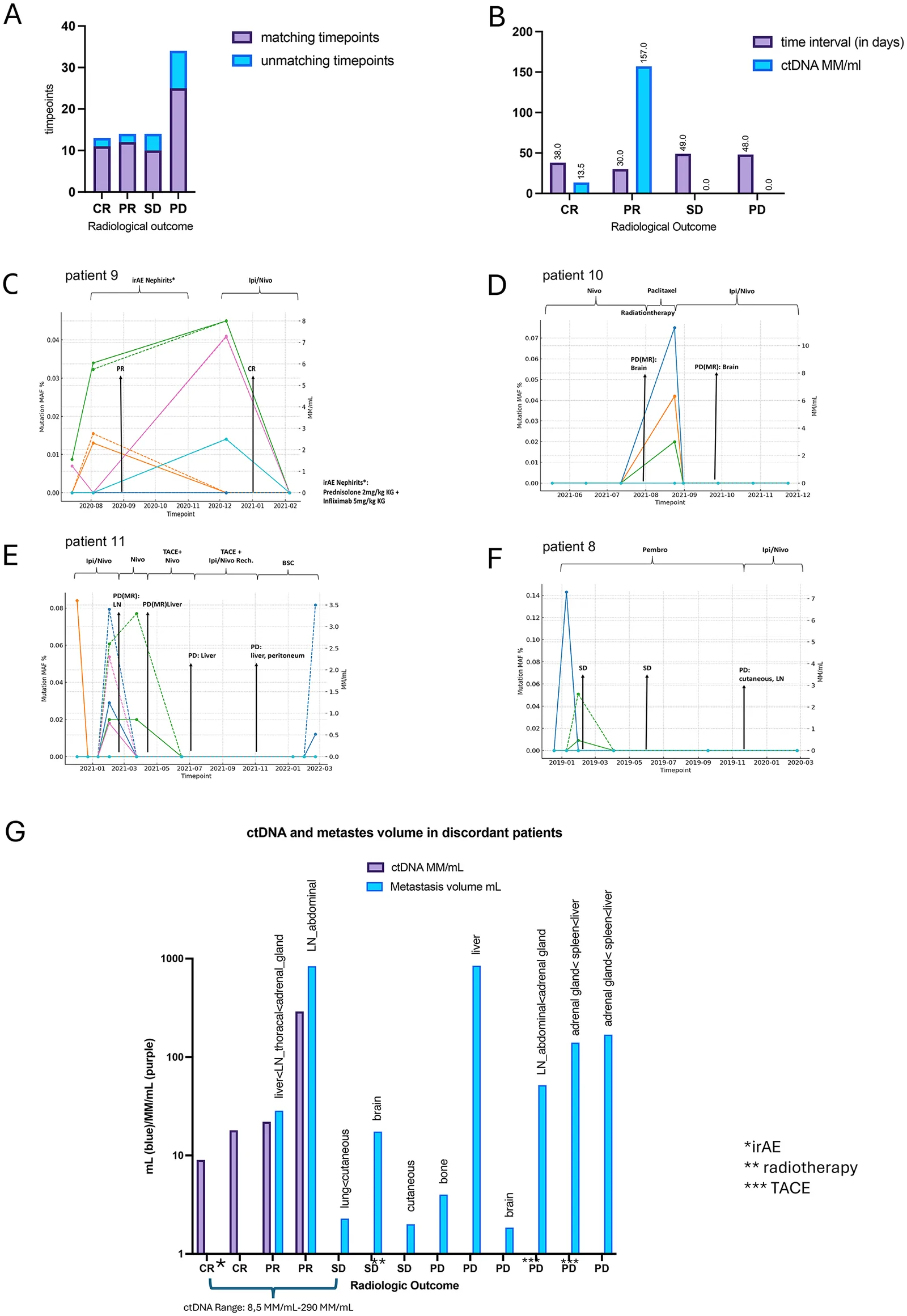
Molecular–radiologic concordance, discordance patterns, and representative discordant ctDNA trajectories. (**A**) Concordance between ctDNA measurements and radiologic response according to RECIST 1.1. Bars indicate the number of matched ctDNA–radiology pairs classified as concordant (purple) or discordant (blue), stratified by radiologic response category (CR, PR,SD, PD). (**B**) Mean ctDNA time intervals (days; purple) relative to radiologic assessment and mean ctDNA concentration (MM/mL; blue) for each radiologic outcome category (CR: 38 days, 13.5 Mm/mL; PR: 30 days, 157.0 MM/mL; SD: 49 days, 0 MM/mL; PD: 48 days, 0 MM/mL). (C–F) representative longitudinal patient cases illustrating common patterns of molecular–radiologic discordance observed during immune checkpoint inhibitor therapy. ctDNA concentrations (Mm/mL) are shown on the left ordinate, while metastatic tumor burden (mL), when available, is displayed on the right ordinate. Treatment phases (e.g., ipilimumab/nivolumab, nivolumab monotherapy, pembrolizumab, paclitaxel, transarterial chemoembolization (TACE), and radiotherapy) are indicated above each timeline. Vertical arrows denote radiologic assessments (CR, PR, SD, PD) and relevant clinical events, including organ-associated disease progression. These representative cases illustrate molecular progression preceding radiologic progression, persistent ctDNA despite radiologic response, and radiologic progression in the absence of detectable ctDNA. (**G**) Comparison of ctDNA concentration (purple bars, MM/mL) and volumetric tumor burden (blue bars, mL; logarithmic scale) at the time of radiologic assessment in representative discordant cases (*n* = 13). Cases are grouped according to radiologic response category and dominant metastatic site. The previously measured ctDNA values averaged 0 MM/mL in PD and SD, 157 MM/mL in PR (range 22 MM/mL − 291 MM/mL), and 14 MM/mL in CR (range 9 MM/mL − 18 MM/mL). Three patients were excluded from this comparison because no contemporaneous imaging was available at the corresponding ctDNA assessment. One additional patient was excluded because the lesion was below the predefined threshold for reliable volumetric quantification. *CR, complete remission; PD, progressive disease; PR, partial remission; SD, stable disease*

Overall concordance between ctDNA dynamics and radiologic response (RECIST 1.1) was high in responding disease states (85.0% [CR/PR]), but substantially lower in SD and PD patients, with 72.9% concordance between ctDNA and consecutive radiologic imaging outcomes (Fig. 5A). Overall, 17 discordant time points from 13 patients were identified (CR *n* = 2; PR *n* = 2; SD *n* = 4; PD *n* = 9).

At the discordant time points, the mean interval was 48 days for PD (range 2–94 days), 49 days for SD (range 0–114), 30 days for PR (range 12–47), and 38 days for CR (range 37–38) (Fig. 5B). Discordant patterns included (i) increasing ctDNA levels (>2 MM/mL) during PR/CR and (ii) persistently low or undetectable ctDNA (<2 MM/mL) during SD or PD. Representative examples of these mismatch patterns are shown in (Figs. 5C–F). Quantitative characterization demonstrated marked heterogeneity across discordant cases. Mean tumor volume ranged from 0 mL in CR to 433.2 mL in PR, whereas preceding ctDNA concentrations ranged from undetectable in PD and SD to 157 MM/mL in PR (Fig. 5G).

Discordant cases were enriched for immune-related adverse events, locoregional therapies (radiotherapy and transarterial chemoembolization (TACE)), and ctDNA-negative metastases, particularly in SD and PD (Fig. 5G). At the time of assessment, one patient with a complete response (CR) and detectable ctDNA experienced an immune-related adverse event (irAE), specifically nephritis. One patient with stable disease (SD) and non-detectable ctDNA was undergoing radiotherapy at the time of measurement. Among patients with progressive disease (PD) and non-detectable ctDNA, two were receiving liver-directed therapy (TACE) at the time of assessment. Additionally, one PD patient with brain metastases was undergoing radiotherapy at the time of measurement (Fig. 5G). A detailed classification of representative discordant cases has been added as Supplementary Table 7, including ctDNA kinetics, volumetric tumor burden, metastatic sites, treatment context, and potential clinical explanations.

## Discussion

Unlike previous studies that primarily evaluated ctDNA in relation to RECIST response or survival, our study integrates longitudinal ctDNA profiling with quantitative volumetric imaging and temporal matching, providing a more comprehensive framework for understanding the biological and methodological determinants of ctDNA dynamics. In this study, we integrated longitudinal ctDNA measurements with RECIST 1.1 response and total metastatic tumor volume, combining static, dynamic, organ-associated, and temporally matched analyses to define the clinical utility and biological limitations of ctDNA for disease monitoring in advanced melanoma. Our findings demonstrate that ctDNA reflects radiologic disease status and total metastatic tumor volume but is substantially influenced by temporal alignment, metastatic site, and biological heterogeneity. Together, these factors explain why ctDNA should be interpreted as a complementary longitudinal biomarker rather than a standalone surrogate of disease burden.

Longitudinal ctDNA profiling revealed marked inter-individual and mutation-specific heterogeneity. In several patients, increases in ctDNA preceded radiologic progression, whereas persistent ctDNA detectability despite radiologic response suggested ongoing residual disease. In addition, asynchronous dynamics among concurrent mutations illustrate ctDNA’s ability to capture clonal evolution that cannot be appreciated by imaging alone. These findings are consistent with previous studies showing that serial ctDNA measurements provide biologically meaningful information beyond conventional radiologic assessment [2, 5, 12, 16–18]. In clinically challenging situations such as mixed response, ctDNA may therefore complement imaging by identifying emerging disease activity before morphologic progression becomes evident. Whether such molecular information can guide treatment decisions and improve patient outcomes remains to be addressed in prospective studies.

Consistent with previous reports, ctDNA concentrations increased with worsening RECIST response and correlated with total tumor volume [19]. However, both associations were moderate rather than strong (overall AUC 0.69, ρ = 0.31, 95% CI 0.20–0.52, *p* = 0.002).

Threshold-based analyses demonstrated a clear sensitivity–specificity trade-off: for example, a threshold of 10 MM/mL yielded a sensitivity of 52% and specificity of 76%, whereas higher thresholds (≥25 MM/mL) increased specificity at the expense of sensitivity. These findings indicate that elevated ctDNA levels are informative for confirming active disease, while low levels are insufficient to reliably exclude progression [2, 7].

The moderate cross-sectional performance of ctDNA likely reflects fundamental biological and methodological differences compared to imaging. Whereas RECIST 1.1 captures static, morphology-based changes in selected lesions, ctDNA represents a dynamic and complex signal influenced by tumor cell turnover and, consequently, shedding of DNA fragments by apoptotic cells - a biological process that is not fully understood yet. This discrepancy is particularly evident in intermediate response categories such as PR and SD, where substantial residual tumor burden or variable baseline disease extent may weaken associations with ctDNA levels. Accordingly, ctDNA appears most informative when interpreted longitudinally rather than as a single time point measurement [19, 20].

In line with this, ctDNA concentrations were positively associated with total tumor volume (ρ = 0.46), with stronger correlations when sampling and imaging were temporally aligned (ρ = 0.56). However, changes in ctDNA were not significantly associated with volumetric changes. This likely reflects nonlinear and lesion-specific shedding dynamics [2, 6, 7], as well as the influence of treatment-related effects such as inflammation, immune cell infiltration, and pseudoprogression, particularly under immunotherapy [18, 21–23]. These findings further underscore that ctDNA reflects dynamic disease activity. Moreover, when blood sampling and imaging are closely synchronized in time, the biological interpretability of ctDNA-tumor burden associations improves substantially [16, 21, 24].

Our organ-associated analyses further highlight the importance of spatial heterogeneity. Associations between ctDNA and total metastatic tumor volume differed substantially across metastatic sites, with stronger correlations observed for lymph node metastases and limited detectability in lung, liver, and particularly brain metastases. These observations are biologically plausible given known differences in vascularization, lymphatic drainage, blood-brain barrier integrity, and cfDNA clearance The liver, for example, plays a central role in cfDNA clearance, which may counterbalance high local ctDNA release, whereas lymph node metastases may facilitate more direct release of tumor-derived DNA via the lymphatic system. Peritoneal metastases showed the highest discriminative performance in ROC analysis (AUC = 0.85, *n* = 7); however, given the comparatively small number of observations, this finding should be interpreted with caution and regarded as hypothesis-generating rather than conclusive.

Notably, in colorectal cancer, ctDNA concentrations are higher in patients with liver metastases than peritoneal metastases [25], which may reflect the different location and biology of the primary tumors. In addition, under immune checkpoint inhibition, increased immune-mediated tumor cell turnover within lymph nodes may further enhance ctDNA release. Because ctDNA shedding presumably varies between organs, it cannot reliably reflect overall tumor burden and should be interpreted alongside disease distribution, especially when considering reducing imaging based on low ctDNA levels.

Volumetric changes in poorly shedding compartments may be underrepresented in ctDNA dynamics, while changes in high-shedding sites disproportionately influence circulating ctDNA levels [26, 27]. In particular, brain metastases pose a unique challenge due to the blood-brain barrier, which restricts the release of tumor-derived DNA into the peripheral circulation [26, 28]. However, because most patients harbored metastases in multiple anatomical compartments, these findings should be interpreted with caution as potential organ-associations rather than direct evidence of organ-associated shedding behavior. Validation in larger cohorts including patients with isolated organ involvement will therefore be required.

Analysis of discordant cases between ctDNA dynamics and radiologic response further illustrates the complexity of ctDNA interpretation. While concordance was high in responding disease (CR/PR: 85.0%), it was lower in stable or progressive disease (72.9%). Patients with rapidly progressing metastatic disease may show low rates of apoptosis and, as a consequence, lower ctDNA levels than expected from the tumor burden [29]. Discordant patterns included rising ctDNA despite radiologic response and, conversely, low or undetectable ctDNA despite SD or PD. In addition to the previously described biological factors, clinical factors such as immune-related adverse events, radiotherapy, and locoregional treatments contributed to discordance. These findings emphasize that ctDNA, similar to established biomarkers such as LDH or S100, must be interpreted within the broader clinical context and should not be used as a single-time-point marker.

Overall, our findings indicate that the clinical value of ctDNA lies in complementing rather than replacing radiologic assessment. ctDNA integrates biological information from multiple metastatic lesions and therefore adds a different perspective on disease activity to the information derived from imaging. The value of ctDNA is therefore most likely achieved through serial longitudinal assessment integrated with imaging, volumetric analyses, and clinical context rather than isolated single time-point measurements.

## Limitations

This study has several limitations. First, temporally matched ctDNA-radiology pairs were analyzed as independent observations without explicitly accounting for repeated measurements within individual patients, potentially leading to an overestimation of statistical significance. Second, the relatively small cohort limited the statistical power of subgroup analyses, particularly the organ-associated analyses, which should therefore be regarded as exploratory and hypothesis-generating. Third, patients received different immune checkpoint inhibitor regimens according to routine clinical practice, and treatment-specific differences in ctDNA kinetics cannot be excluded. Fourth, the targeted sequencing panel was restricted to hotspot mutations in BRAF, NRAS, KRAS, EGFR, and PIK3CA, potentially reducing ctDNA detectability in patients harboring other melanoma-associated alterations such as TERT promoter or KIT mutations. Fifth, the predefined ±40-day window used to match ctDNA sampling with radiological imaging in the primary analyses reflects real-world clinical scheduling but permits temporal discrepancies that may attenuate the biological relevance of the observed associations; our temporally stratified sensitivity analyses showed that the most robust association was observed when sampling and imaging were performed within 30 days, whereas correlations at longer intervals were weaker or limited by small sample sizes, underscoring the importance of close temporal alignment for accurately capturing the dynamic relationship between ctDNA and total metastatic tumor volume. Finally, lesions smaller than 0.5 mL were excluded from volumetric analyses because of limited segmentation reliability, although these represented only a small proportion of the total metastatic burden.

## Conclusion

In this study, ctDNA emerged as a biologically informative marker that reflects overall tumor burden and disease activity, while also capturing aspects of tumor biology that are not fully represented by conventional imaging. Our findings support the potential clinical utility of ctDNA as a complementary biomarker for longitudinal disease monitoring and molecular characterization in advanced melanoma. Accordingly, ctDNA should be regarded as a complementary biomarker that provides additional information when interpreted alongside imaging findings and clinical assessment rather than as an independent surrogate of disease burden. ctDNA levels are influenced by tumor distribution, organ-associated differences in ctDNA shedding, ongoing therapies such as radiotherapy or locoregional interventions, and patient-specific factors that affect cfDNA dynamics.

The integration of ctDNA into clinical workflows, therefore, requires more than analytical validation.Prospective studies are needed to determine whether ctDNA-guided treatment strategies can improve patient outcomes and to define standardized frameworks for its integration into clinical practice.

## Electronic supplementary material

Below is the link to the electronic supplementary material.

Supplementary Material 1

Supplementary Material 2

## Data Availability

The majority of the data generated or analyzed during this study are included in this published article and its supplementary information files. Part of the data generated in this study is not publicly available due to patient privacy requirements, but is available upon reasonable request to the corresponding author.

## Abbreviations

ctDNA: Circulating tumor DNA
UMI: Unique Molecular Identifier
ICI: Immune Checkpoint Inhibition
LB: Liquid biopsy
PCR: Polymerase chain reaction
NGS: Next-generation sequencing
CTLA-4: Cytotoxic T-lymphocyte-associated protein 4
PD-1: Programmed cell death protein 1
AJCC: American Joint Committee on Cancer
T: Tumor
N: Nodes
M: Metastases
IQR: Interquartile range
SD: Standard deviation
MM: Mutant molecules
CDS: Coding DNA Sequence
AAC: Amino Acid Changes
LDH: Lactate dehydrogenase
S100: S100 protein
RECIST: Response Evaluation Criteria in Solid Tumors
MRI: Magnetic resonance imaging
CT: Computer tomography
PD: Progressive disease
MR: Mixed response
SD: Stable disease
PR: Partial remission
CR: Complete remission
LN: Lymph node
PFS: Progression-free survival
Human Genes; BRAF: B-Raf proto-oncogene
KRAS: Kirsten rat sarcoma virus
EGFR: Epidermal growth factor receptor
PIK3CA: Phosphatidylinositol-4,5-bisphosphate 3-kinase cata- lytic subunit alpha
NRAS: neuroblastoma RAS viral proto-oncogene
