# Supplementary material for: Integrated longitudinal analysis of ctDNA, radiologic response, and tumor volume reveals spatial and temporal heterogeneity in advanced melanoma
Source: J Transl Med. 2026 Sep 17;24:1199. doi: 10.1186/s12967-026-08899-0 (PMC13589358; doi:10.1186/s12967-026-08899-0)
Supplement: Supplementary file 1 [file 12967_2026_8899_MOESM1_ESM.docx]

**Supplementary Table 1.** **Association of baseline LDH and S100 concentrations with baseline ctDNA status**

| ctDNA at baseline | Total N |  | Negative | Positive | p |
| --- | --- | --- | --- | --- | --- |
| Baseline LDH (U/L) | 42 (100.0) | Mean (SD) | 270.353 (106.730) | 312.120 (124.715) | 0.2526 |
| Baseline S100B (µg/L) | 42 (100.0) | Mean (SD) | 0.162 (0.179) | 0.660 (0.893) | **0.0116** |

*ctDNA, circulating tumor DNA; LDH, lactate dehydrogenase; S100, S100 calcium-binding protein B; SD, standard deviation.*

**Supplementary Table 2.** **Univariable and multivariable Cox regression analysis of overall survival**

| Overall Survival Cox Regression |  |  | HR (univariable) | HR (multivariable) |
| --- | --- | --- | --- | --- |
| ctDNA at baseline | Negative | 17 (40.5) | - | - |
|  | Positive | 25 (59.5) | 1.30 (0.51-3.30, p=0.582) | - |
| LDH at baseline | Not elevated | 19 (45.2) | - | - |
|  | Elevated | 23 (54.8) | 1.42 (0.56-3.62, p=0.458) | - |
| S100B at baseline | Not elevated | 22 (52.4) | - | - |
|  | Elevated | 20 (47.6) | 1.78 (0.71-4.43, p=0.216) | - |

*HR, hazard ratio; CI, confidence interval; ctDNA, circulating tumor DNA; LDH, lactate dehydrogenase; S100, S100 calcium-binding protein B.*

**Supplementary Table 3.** **Univariable and multivariable Cox regression analysis of progression-free survival**

| Progression-free survival Cox Regression |  |  | HR (univariable) | HR (multivariable) |
| --- | --- | --- | --- | --- |
| ctDNA at baseline | Negative | 17 (40.5) | - | - |
|  | Positive | 25 (59.5) | **2.53 (1.13-5.64, p=0.024)** | 2.24 (0.97-5.15, p=0.058) |
| LDH at baseline | Not elevated | 19 (45.2) | - | - |
|  | Elevated | 23 (54.8) | 1.27 (0.60-2.69, p=0.531) | - |
| S100B at baseline | Not elevated | 22 (52.4) | - | - |
|  | Elevated | 20 (47.6) | 1.66 (0.79-3.51, p=0.183) | - |
|  |  |  |  |  |
|  |  |  |  |  |

***HR****, hazard ratio;* ***CI****, confidence interval;* ***ctDNA****, circulating tumor DNA;* ***LDH****, lactate dehydrogenase;* ***S100****, S100 calcium-binding protein B;* ***PFS****, progression-free survival.*

**Supplementary Table 4. Influence of lead time between ctDNA measurement and radiological imaging**

*CI, confidence interval; CR, complete remission; PR, partial remission; SD, stable disease; PD, progressive disease*

**Supplementary Table 5.**

**Influence of Leadtime between ctDNA measurement and volume measurement**

| **Lead time** | **N** | **Spearman r** | **95% CI** | **P value (two sided)** | **Significant (alpha=0.05)** |
| --- | --- | --- | --- | --- | --- |
| **0–30 days** | 42 | 0.5639 | 0.3–0.75 | < 0.0001 | yes |
| **30–90 days** | 24 | 0.2320 | 0.08-0.49 | 0.29 | no |
| **> 90 days** | 8 | 0.5816 | 0.41-0.8 | 0.18 | no |

*CI, confidence interval*

**Supplementary Table 6. Influence of** Δ**ctDNA and** Δ**Volume**

CI, confidence interval

**Supplementary Table 7.** **Classification of representative molecular–radiologic discordance cases**

| **RECIST 1.1** | **Leadtime ctDNA-radiology (days)** | **Additional treatment** | **irAE at timepoint** | **ctDNA MM/mL** | **Metastasis volume (mL)** | **Origin of metastasis** |
| --- | --- | --- | --- | --- | --- | --- |
| CR | 37 |  | Nephritis | 9 | 0 |  |
| CR | 38 |  |  | 18 | 0 |  |
| PR | 47 |  |  | 291 | 837.88 | Liver < thoracic LN < adrenal gland |
| PR | 12 |  |  | 22 | 28.53 | Abdominal LN |
| SD | 114 |  |  | 0 | NA | Cutaneous |
| SD | 5 | Radiotherapy |  | 0 | 2.28 | Lung < cutaneous |
| SD | 0 |  |  | 0 | 17.5 | Brain |
| PD | 27 |  |  | 0 | Not measurable | Cutaneous |
| PD | 28 | Liver directed treatment (TACE) |  | 0 | 846.4 | Liver |
| PD | 148 | Liver directed treatment (TACE) |  | 0 | NA | Cutaneous< bone <LN |
| PD | 2 | Radiotherapy |  | 0 | 1.85 | Brain |
| PD | 23 |  |  | 0 | 51.78 | Abdominal LN <adrenal gland |
| SD | 77 |  |  | 0 | 2 | Cutaneous |
| PD | 84 |  |  | 0 | 4 | Bone |
| PD | 94 |  |  | 0 | NA | LN |
| PD | 34 |  |  | 0 | 140.2 | Adrenal gland< spleen<liver |
| PD | 7 |  |  | 0 | 169.07 | Adrenal gland< spleen<liver |

*ctDNA, circulating tumor DNA; RECIST, Response Evaluation Criteria in Solid Tumors; CR, complete response; PR, partial response; SD, stable disease; PD, progressive disease; MM/mL, mutant molecules per milliliter; TACE, transarterial chemoembolization; irAE, immune-related adverse event; LN, lymph node.*
