# Supplementary material for: Integrated longitudinal analysis of ctDNA, radiologic response, and tumor volume reveals spatial and temporal heterogeneity in advanced melanoma
Source: J Transl Med. 2026 Sep 17;24:1199. doi: 10.1186/s12967-026-08899-0 (PMC13589358; doi:10.1186/s12967-026-08899-0)
Supplement: Supplementary file 2 [file 12967_2026_8899_MOESM2_ESM.docx]

**Supplementary Figure 1.** Illustrative Examples of Three-Dimensional Tumor Segmentation and Volumetric Assessment.

**(A)-(D):** Representative example images illustrating the volumetric assessment of total tumor burden across all metastases. Tumor volumes were quantified using Sectra IDS7 software.

**Supplementary Figure 2.** **Longitudinal patient-specific trajectories of ctDNA, tumor burden, and radiologic response**. Longitudinal ctDNA profiles of 42 representative patients illustrating distinct molecular–radiologic response patterns during treatment. For each panel, mutation-specific ctDNA allele frequencies (MAF, %, left ordinate) and absolute ctDNA concentrations (MM/mL, right ordinate) for *BRAF, EGFR, KRAS, NRAS,* and *PIK3CA* are shown over time. Radiologic staging events (PR, CR, SD, PD) are indicated by vertical arrows. Treatment regimens are annotated above each panel.

**Supplementary Figure 3.** **ctDNA levels and tumor volume stratified by number of metastases**. Boxplots show circulating tumor DNA (ctDNA) levels (MM/mL) and total tumor volume in patients with 1–3 metastases compared to patients with >3 metastases. ctDNA levels are presented as mutant molecules per milliliter (MM/mL), and tumor volume was derived from three-dimensional segmentation of all measurable lesions. Patients with >3 metastases exhibited significantly higher tumor volumes compared to those with 1–3 metastases (Mann–Whitney U test, p = 0.017). In contrast, ctDNA levels did not differ significantly between groups (p = 1.00), despite higher mean values observed in patients with fewer metastases.
Boxes represent the interquartile range (IQR), with the median indicated by a horizontal line. Whiskers extend to the minimum and maximum values.
